# The tactics of respiratory therapy after palliative anastomosis fallot's tetralogy patients with hypervolemia lesser circulation during intensive management

**DOI:** 10.1186/2197-425X-3-S1-A951

**Published:** 2015-10-01

**Authors:** RA Ibadov, HK Abrolov, NA Strijkov, DI Zhulamanova

**Affiliations:** Republican Specialized Center of Surgery Named after Acad. V. Vakhidov, Tashkent, Uzbekistan

## The Aim

optimization of respiratory tactics of early postoperative period after regulated subclavian-pulmonary anastomosis in patients with Fallot's tetralogy and lesser circulation.

## Materials and Methods

81 patients with and lesser circulation after elective surgery regulated subclavian-pulmonary anastomosis for the last 2 years, in the ICU RSCS after V.Vakhidov. Proximal end of tourniquet regulators was used in case of 39 patients and Fogarty catheter for volume dosing of shunt was used in case of 42 patients. Clinical examination: EchoCG (with detection of velocity of blood flowing through the subclavian-pulmonary regulated anastomosis), ECG, chest X-rays (with special emphasis on assessing the degree of blood filling in the pulmonary circulation); cardiac monitoring: heart rate, blood pressure, central venous pressure, gas exchange parameters and deep oxygen status (pH, SpO_2_, pO_2_, pCO_2_, lactate); monitoring of hemoglobin, hematocrit levels and data of blood coagulation.

## Results

From overall patients 14 patients had hyperfunction of anastomosis: the picture of pulmonary preedema was noted in 6 cases, and 9 patients had “managed” hypervolemia of the pulmonary circulation that manifested as: hemodynamic instability (MABP 75-80 mm Hg, HR-120-140 bpm, CVP - 100-140 mm H_2_O), increase of SpO_2_ to 90,1 ± 1,2 together with poor values of deep oxygen status (A-a-205,1 ± 5,3 mm Hg, a/A-47,3% ± 1,4). Restriction of anastomosis functioning allowed to achieve stabilization of hemodynamic (blood pressure 90 - 100 mm Hg), deep oxygen status (A-a and -230 mm Hg, a/A-30%, SpO_2_- 80% at FiO2- 40%), which were accompanied by disappearance of rales, improvement of echocardiographic data in dynamics with reduced duration of mechanical ventilation and time of staying in the ICU. Approximate initial date MLV: Vt = 7-9 ml/kg, f = 15-17, I:E = 1:2, PEEP = 5 sm H_2_O, FiO2 = 40-50%, trigger = 3-3,5L/min or 2,5-3 sm H_2_O. On reaching satisfactory performance of gases of blood, stable haemodynamics, good functioning SPA and good X-ray picture patients expose to extubation.

## Conclusions

The proposed tactics of intensive care in the early postoperative period after controlled subclavian-pulmonary anastomosis in patients with Fallot's tetralogy allows monitoring and active controlling of the volume of shunted through the anastomosis blood, thus helping to avoid development of hyperfunctioning of anastomosis and pulmonary edema.

